# Web-Based Interventions Supporting Adolescents and Young People With Depressive Symptoms: Systematic Review and Meta-Analysis

**DOI:** 10.2196/mhealth.8624

**Published:** 2017-12-08

**Authors:** Maritta Välimäki, Katriina Anttila, Minna Anttila, Mari Lahti

**Affiliations:** ^1^ Hong Kong Polytechnic University Hong Kong China; ^2^ Department of Nursing Science University of Turku Turku Finland; ^3^ Turku University Hospital Turku Finland; ^4^ Division of Child Psychiatry Helsinki University Hospital Helsinki Finland; ^5^ Turku University of Applied Science Turku Finland

**Keywords:** Internet, adolescent, depression, meta-analysis, information and communication technology, intervention, systematic review, treatment as usual

## Abstract

**Background:**

Although previous studies on information and communication technology (ICT)–based intervention on mental health among adolescents with depressive symptoms have already been combined in a number of systematic reviews, coherent information is still missing about interventions used, participants’ engagement of these interventions, and how these interventions work.

**Objective:**

We conducted a systematic review and meta-analysis of trials to describe the effectiveness of Web-based interventions to support adolescents with depression or depressive symptoms, anxiety, and stress. We also explored the content of the interventions, as there has previously been a lack of coherent understanding of the detailed content of the Web-based interventions for these purposes.

**Methods:**

We included parallel randomized controlled trials targeted at adolescents, or young people in the age range of 10 and 24 years, with symptoms or diagnoses of depression and anxiety. The interventions were from original studies aimed to support mental health among adolescents, and they were delivered via Web-based information and communication technology.

**Results:**

Out of 2087 records identified, 27 papers (22 studies) met the inclusion criteria. On the basis of a narrative analysis of 22 studies, a variety of Web-based interventions were found; the most commonly used intervention was based on cognitive behavioral therapy. Meta-analysis was further conducted with 15 studies (4979 participants). At the end of the intervention, a statistically significant improvement was found in the intervention group (10 studies) regarding depressive symptoms (*P*=.02, median 1.68, 95% CI 3.11-0.25) and after 6 months (3 studies; *P*=.01, median 1.78, 95% CI 3.20-0.37). Anxiety symptoms (8 studies; *P*<.001, median 1.47, 95% CI 2.36-0.59) and moods and feelings (2 studies; *P*=.04, median 5.55, 95% CI 10.88-0.22) improved as well in the Web-based intervention group, but there was no difference in stress scores. However, adolescents in the intervention group left the study early more often, both in short-term studies (11 studies; *P*=.007, median 1.31, 95% CI 1.08-1.58) and mid-term studies (3 studies; *P*=.02, median 1.65, 95% CI 1.09-2.49). We did not find any studies that had assessed the costs of the Web-based interventions.

**Conclusions:**

Despite widely reported promises that information technology use is beneficial to adolescents with depression, the results of our review show only short-term effects on adolescents’ mental well-being, whereas long-term effects remain questionable because of the limited number of studies reviewed. Information about the economic benefits of Web-based interventions is still lacking. The quality of the studies, especially biases related to attrition rates and selective reporting, still needs serious attention.

## Introduction

Currently, about half of adolescents showing signs of depression get treatment [1]. Identification of potentially effective interventions for adolescents with depression and anxiety is therefore a vital step toward supporting societies in general [2]. Information and communication technology (ICT)–based interventions have the potential to address treatment gaps concerning a variety of mental disorders [3,4]. Over 90% of adolescents use the Internet daily and 56% several times a day [5]. The Internet allows anonymous participation [4], without the fear of stigmatization [6]. Other benefits may include cost-effectiveness [7,8] and high accessibility [3].

Although a wide range of ICT-based interventions has been developed and tested, the impact of these interventions is still controversial in the field of mental health. On the basis of previous reviews, cognitive behavioral therapy (CBT)–based Web-based interventions have been found to impact the appearance of depressive and anxiety symptoms among young people [9-11], whereas online and mobile psychosocial suicide prevention intervention has reduced suicidal ideation, depression, and hopelessness [12]. A relevant study by Reyes-Portillo et al [13] reviews the effectiveness of Web-based treatment and prevention interventions developed for anxiety, depression, and suicide prevention. They found that 10 out of the 25 studies they reviewed reported significant postintervention reductions in symptoms, or improvements in diagnostic ratings. However, the evidence supporting the effectiveness of Internet-based interventions for youth depression and anxiety is still limited. Ye et al [14] performed a meta-analysis on 7 studies related to these types of interventions for young people. They observed a decrease in the severity of anxiety symptoms, but not a statistically significant decrease in depressive symptoms, when the results were compared with a wait list group. Nor were statistical differences found in depressive symptoms when Internet-based treatment was compared with face-to-face treatment in 2 studies. Furthermore, Kauer et al [15] did not find any improvement in the behavior among young people when it came to seeking help from Web-based services (18 studies), and a narrative review by Best et al [16] on the effects of social media technology on adolescent well-being found mixed effects or no effects at all.

Concerns regarding these Web-based intervention studies include methodological flaws such as heterogeneity in the interventions in terms of content, settings, dose, or quality [9,17]. A review by Arnberg et al [18] showed that the quality of evidence was graded as low or very low, and therefore, no conclusions were able to be drawn. Concerns also include insufficient search processes of the literature [10], small sample sizes [13,15], and differences in baseline in study samples [14]. A publication bias toward positive results has also been expressed [10,11,13,18].

Despite promising results of ICT-based interventions for adolescents and young people with depression, the overall picture of the effectiveness of these interventions is still inconclusive. To understand how the intervention works [9], we need to consider in more detail the content and structure of the interventions [19]. Therefore, in this systematic review, we describe the Web-based interventions and explore the impact of these interventions on the reduction of depressive symptoms among adolescents and young people with symptoms or a diagnosis of depression.

## Methods

The methods of this systematic review have been based on the preferred reporting items for systematic reviews and meta-analysis (PRISMA) [20]. PRISMA-P for meta-analysis protocols [21] and Cochrane handbook for systematic reviews of interventions [22] were also used in the preparation of our meta-analysis. Where possible, the data extraction was based on the CONSORT-EHEALTH checklist version 1.6.1 [23]. Web-based interventions were described using the template for intervention description and replication (TIDieR) checklist and guide [24].

### Eligibility Criteria

The review was limited to assessing the effectiveness of the interventions using a randomized controlled trial (RCT) design to gather only high-quality studies about health care interventions [25]. We included studies targeted at adolescents or young people in the age range of 10 and 24 years [26] who had been diagnosed with depression or had experienced symptoms of depression or anxiety [27]. We focused on interventions that aimed to support mental health among adolescents by preventing, identifying, or decreasing the symptoms of depression or anxiety, or through counseling. The interventions were delivered via ICT, including Web-based technology [28], which could be accessed by computers, tablets, or mobile phones. The review focused on published (or in-press) articles written in peer reviewed journals and published in English. The primary outcome used was depression, and the secondary outcomes used were anxiety, stress, moods and feelings, leaving the study early (attrition rate), and costs.

We excluded dissertations, letters, editorials, literature reviews, book reviews, and book chapters, in addition to studies with designs other than RCT. Study protocols of specific studies were searched for manually and used to verify possible risk of biases. Studies were excluded if the intervention was targeted at adults or persons under 10 or over 25 years old, parents, teachers, or health care staff. If the primary focus of the intervention was something other than depression, such as brain injury, eating disorder, or epilepsy, or if the intervention only included texting, the study was excluded.

### Literature Search Strategy

We conducted a comprehensive literature search on September 1, 2015, and an updated search was done on February 10, 2017. Four electronic databases covering published research from the health and social field were investigated: MEDLINE, PsycINFO, CINAHL, and Cochrane. A combination of medical subject headings and text-based search terms [29] for the databases was used. The search was conducted with the help of an information specialist. Due to changes in user interface in databases between the original and updated search, a search for MEDLINE, PsycInfo, and Cochrane was combined via Ovid (in 2017). Electronic databases used, search terms, and number of hits are documented in Multimedia Appendix 1. For additional references, we consulted the references in the included studies. Relevant systematic reviews were identified through electronic searches to avoid overlap between previous studies.

### Study Selection

**Figure 1:**
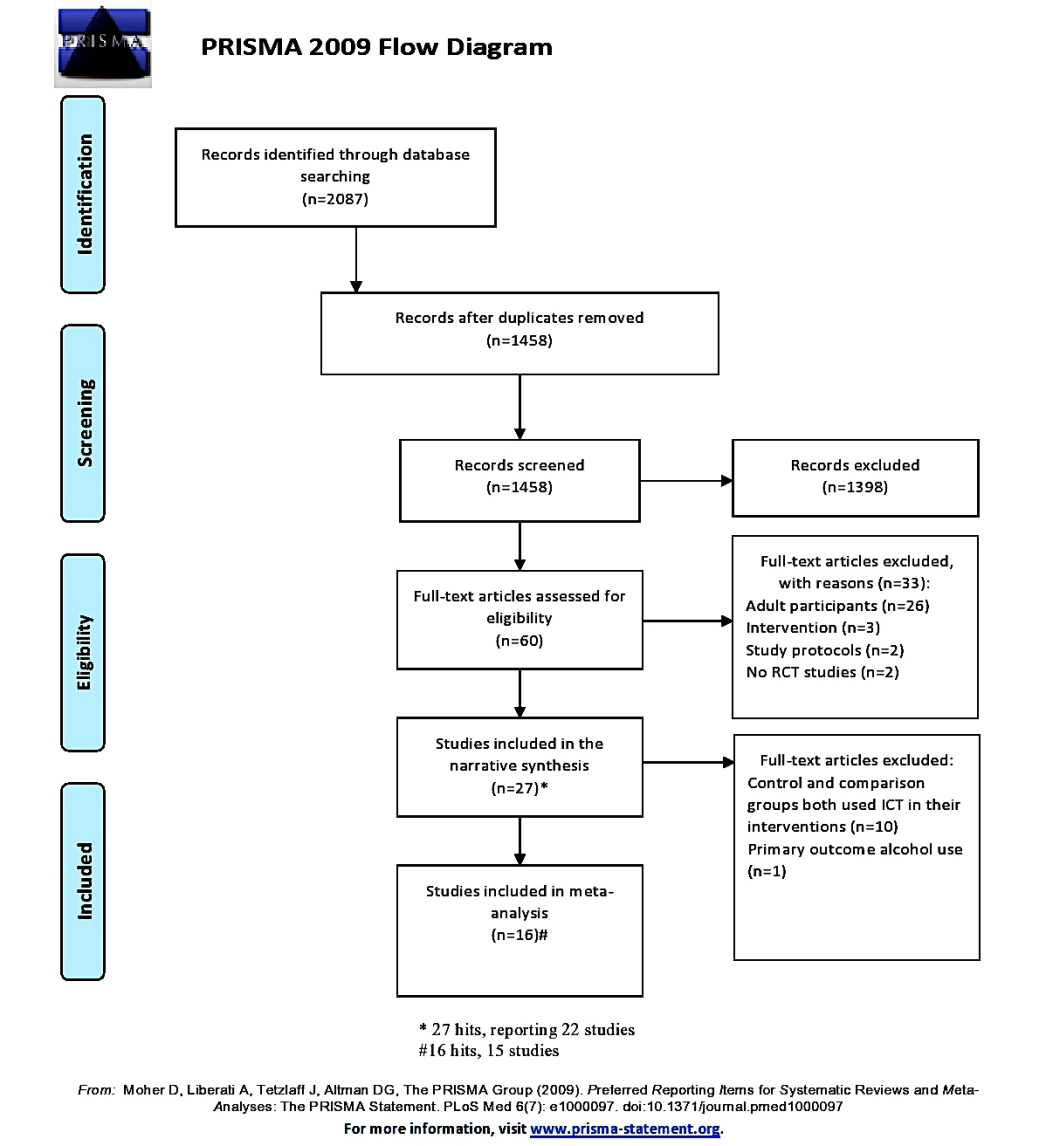
Preferred reporting items for systematic reviews and meta-analysis (PRISMA) flow diagram.

When choosing the selection of studies [22], first, two authors (KA, MA) independently screened all titles of abstracts that were relevant to this systematic review. Second, the abstracts were screened for eligibility. Third, the full papers of the included abstracts were screened for inclusion and exclusion criteria. In cases of discrepancy, the papers were discussed with MV until a consensus was reached. On the basis of the assessment process, the abstracts were included first in the narrative synthesis and later in the meta-analysis (see Figure 1) based on specific criteria, which are recorded in Table 1.

### Data Extraction

We created the data extraction table matrix to collect and describe information, focusing our aims in the synthesis. Data extraction involved describing the included studies and interventions, as well as the excluded studies. For the descriptions of the included studies, the information was collated by authors, year of publication, country of origin, purpose of the study, setting, target group, age, total number of participants randomized, and the number allocated in each study group. Information was combined if the publication (a hit) was based on original data published in more than one paper and the identification was based on the study protocol number. These studies were described in the data extraction tables (matrixes) as one study. The data from 22 included studies (27 hits) for narrative analysis were entered into the specific data extraction grid, where each study was treated as a separate case, and descriptive characteristics of the studies were categorized manually (see Multimedia Appendix 2).

The interventions were extracted to a matrix table based on the TIDieR checklist and guide [24] (see Multimedia Appendix 3). The categorization was done under the following themes based on the thematic analysis of Braun and Clarke [41]: (1) materials and procedures; (2) provider and modes of delivery; (3) location, dose, and length of the program; and (4) tailoring of the intervention, modifications, and fidelity.

### Data Analysis

For the meta-analysis, a summary of outcome measures used in 15 studies is described in Table 2. The meta-analysis was undertaken using Review Manager RevMan version 5.3 (Nordic Cochrane Centre, Cochrane Collaboration, 2014) for preparing and maintaining Cochrane reviews [42]. For continuous outcomes, mean differences were compared between groups. When similar scales were used, presuming that there would be only small differences in measurement tools, measurements were combined. This decision was made to answer the overall question of whether there is evidence that Web-based interventions can be an effective intervention among young people with depression or depressive symptoms [22]. Standard deviations were used with the sample sizes to compute the weight given to each study. A random-effects analysis was used instead of a fixed-effect method, as the former allows the outcomes of studies to vary more than the latter does; a random-effects analysis can be seen as a more natural way of explaining outcomes [43].

In cases of missing or incomplete data, there was an attempt to contact the authors of the study in question. However, as no replies were received from these attempts, available data was used. Heterogeneity was assessed by calculating the I^2^ index. If the estimated I^2^ was greater than or equal to 50%, it was interpreted as indicating the presence of high levels of heterogeneity [22].

### Assessment of the Studies Included in Meta-Analysis

**Figure 2:**
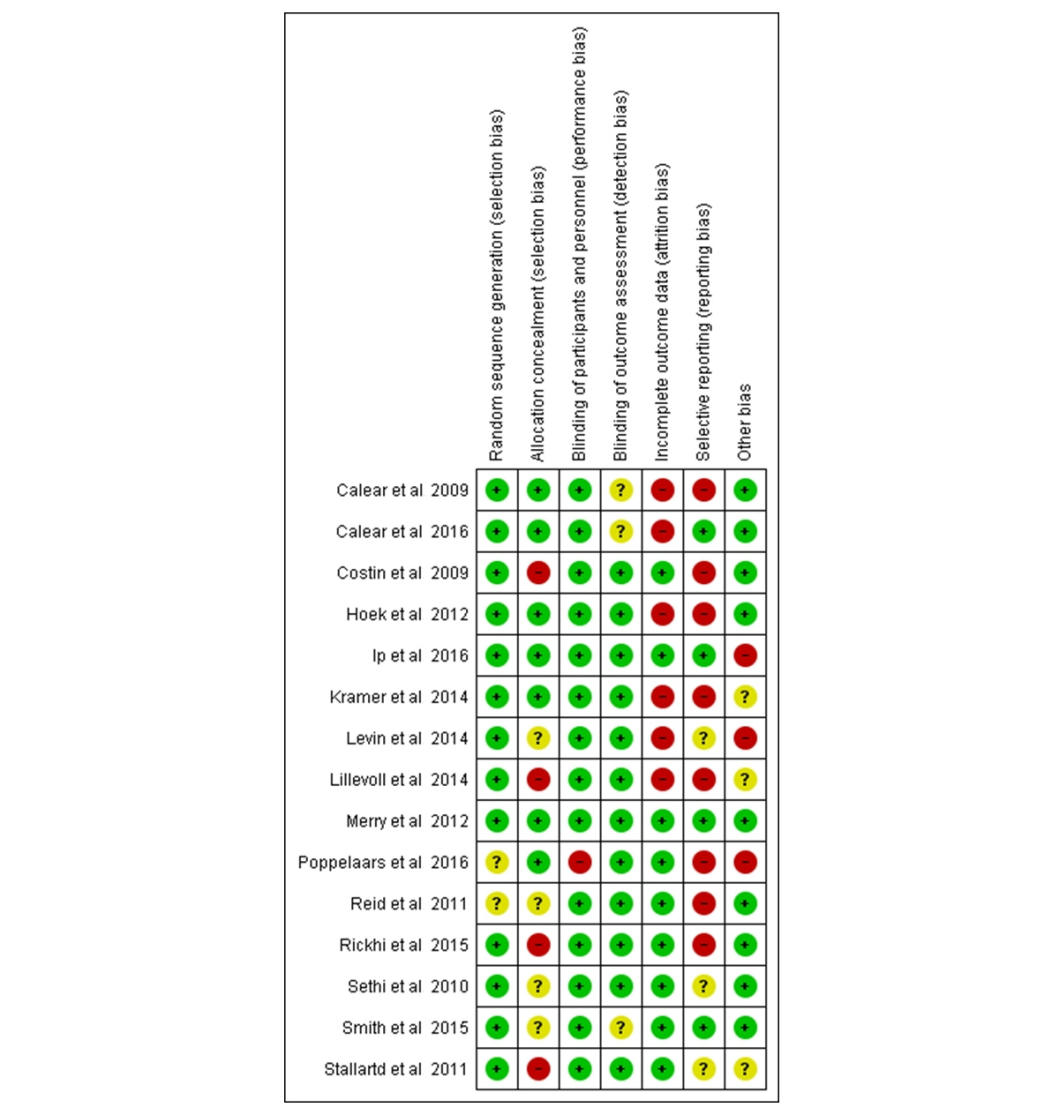
Risk of bias of studies included in the meta-analysis.

The quality of the 15 studies included in the meta-analysis was appraised by using a tool measuring risk of bias from Review Manager (RevMan) version 5.3 [60] with the following criteria [22]: random sequence generation, allocation concealment, blinding of participants and personnel, blinding of outcome assessment, incomplete outcome data, selective reporting, and other bias (Figure 2). For each study, KA and MV classified the domain as having a low, high, or unclear risk of bias. To minimize the risk of publication bias, bibliographic databases and trial registries were consulted to compare original review plans and outcomes reported. Any discrepancy between the two review authors was resolved through discussion (KA, MV, ML).

A sensitivity analysis was conducted by excluding studies (1) with a sample size that vastly differed from other studies or (2) if one or more of the studies had low-quality issues affecting the study results.

**Table 1:** Excluded studies from the meta-analysis and reason for being excluded.

Study	Title
Burckhardt et al 2015 [30]	A Web-based adolescent positive psychology program in schools: a randomized controlled trial^a^
Geisner et al 2015 [31]	Brief Web-based intervention for college students with comorbid risky alcohol use and depressed mood: Does it work and for whom?^b^
Hoek et al 2011 [32]	Randomized controlled trial of primary care physician motivational interviewing versus brief advice to engage adolescents with an Internet-based depression prevention intervention: 6-month outcomes and predictors of improvement^a^
Manicavasagar et al 2014 [33]	Feasibility and effectiveness of a Web-based positive psychology program for youth mental health: randomized controlled trial^a^
Saulsberry et al 2012 [34]	Randomized clinical trial of a primary care Internet-based intervention to prevent adolescent depression: 1 year outcomes^a^
Stasiak et al 2014 [35]	A pilot double blind randomized placebo controlled trial of a prototype computer-based cognitive behavioral therapy program for adolescents with symptoms of depression^a^
Van Voorhees et al 2008 [36]	Integrative Internet-based depression prevention for adolescents: A randomized clinical trial in primary care for vulnerability and protective factors^a^
Van Voorhees et al 2009a [37]	Adolescents dose and rating of an Internet-based depression prevention program: a randomized trial of primary care physician brief advice versus a motivational interview^a^
Van Voorhees et al 2009b [38]	Randomized clinical trial of an Internet-based depression prevention program for adolescents (Project CATCH-IT) in primary care: 12-week outcomes^a^
Whittaker et al 2012 [39]	MEMO—A mobile phone depression prevention intervention for adolescents: development process and postprogram findings on acceptability from a randomized controlled trial^a^
Wright et al 2017 [40]	Computerized cognitive behavioral therapy for depression in adolescents: feasibility results and 4-month outcomes of a UK randomized controlled trial

^a^Intervention: comparison by ICT versus ICT.

^b^Target population: risky alcohol use.

**Table 2:** Authors, outcomes, and outcome measures included in the meta-analysis.

Author (year), protocol number	Depression	Anxiety	Stress	Life satisfaction or quality of life	Moods and thoughts	Leaving the study early	Costs
Calear et al 2009 [44] 2013 [45], ISRCTN67189839	Center for Epidemiological Studies Depression Scale (CES-D)	The Revised Children’s Manifest Anxiety Scale	Not applicable (N/A)^a^	N/A^a^	N/A^a^	Data available	N/A^a^
Calear et al 2016 [46], Published study protocol not available	CES-D^b^	N/A^a^	N/A^a^	N/A^a^	N/A^a^	Data available	N/A^a^
Costin et al 2009 [47], ISRCTN98406912	Symptoms of depression (CES-D)	N/A^a^	N/A^a^	N/A^a^	N/A^a^	Data available	N/A^a^
Hoek et al 2012 [48], NTR1322	CES-D	Hospital Anxiety and Depression Scale	N/A^a^	Client Satisfaction Questionnaire	N/A^a^	Data available	N/A^a^
Ip et al 2016 [49], Published study protocol not available	Center for Epidemiological Studies Depression Scale-Revised (CESD-R) Depression scale (DASS^c^)	Anxiety scale (DASS)	Stress Scale (DASS)	N/A^a^	N/A^a^	Data available	N/A^a^
Kramer et al 2014 [50], NTR1696	Symptoms of depression (CES-D)	N/A^a^	N/A^a^	N/A^a^	N/A^a^	Data available	N/A^a^
Levin et al 2014 [51], Published study protocol not available	Depression scale (DASS)	Anxiety scale (DASS)	Stress scale (DASS)	N/A^a^	N/A^a^	Data available	N/A^a^
Lillevoll et al 2014 [52], Published study protocol not available	N/A^a^	N/A^a^	N/A^a^	N/A^a^	N/A^a^	Data available	N/A^a^
Merry et al 2012 [53], ACTRN12609000249257	Children’s Depression Rating Scale-Revised Reynolds Adolescent Depression Scale-2nd edition (RADS-2)	Spence Children’s Anxiety Scale	N/A^a^	Pediatrics Quality of Life and Satisfaction Questionnaire	Mood and Feelings Questionnaire (MFQ) Hopelessness Scale	Data available	N/A^a^
Poppelaars et al 2016 [54], Published study protocol not available	RADS-2	N/A^a^	N/A^a^	N/A^a^	N/A^a^	Data available	N/A^a^
Reid et al 2011 [55], NCT00794222	Depression scale (DASS)	Anxiety scale (DASS)	Stress scale (DASS)	N/A^a^	N/A^a^	Data available	N/A^a^
Rickhi et al 2015 [56], Published study protocol not available	CDRS-R^b^ Hamilton Depression Rating Scale^b^	N/A^a^	N/A^a^	N/A^a^	N/A^a^	Data available	N/A^a^
Sethi et al 2010 [57], Published study protocol not available	Depression scale (DASS-21) Kessler Psychological Distress Scale	Anxiety scale (DASS-21)	Stress Scale (DASS-21)	N/A^a^	Automatic Thoughts Questionnaire	Data available	N/A^a^
Smith et al 2015 [58], Published study protocol not available	Children’s Response Styles Questionnaire^b^	Screen for Child Anxiety Related Emotional Disorders	N/A^a^	N/A^a^	MFQ	Data available	N/A^a^
Stallard et al 2011 [59], Published study protocol not available	The Adolescent Well-being Scale^b^	The Spence Children’s Anxiety Scale child version^b^	N/A^a^	N/A^a^	The Schema Questionnaire for Children	Data available	N/A^a^

^a^N/A signifies missing outcome.

^b^Usable data not available.

^c^DASS: Depression Anxiety Stress Scales.

## Results

### Results of the Search

We screened 2087 hits of abstracts, which were identified through electronic databases. On the basis of the screening, we removed 1458 duplicates. After assessing their eligibility, 1398 abstracts were excluded, which left us with 60 abstracts. Sixty full papers were then retrieved for potential inclusion in the study, and their eligibility was assessed based on our inclusion and exclusion criteria. We excluded 33 abstracts, which left us with 27 paper hits (22 studies) included in the narrative synthesis. Overall, 16 paper hits (15 studies) were included in the meta-analysis (see Figure 1).

### Characteristics of the Studies

#### Narrative Analysis

The studies included in the narrative analysis (22 studies, 27 papers) were published from 2008 to 2017. They were conducted in school environments, health care settings, or in community settings in Australia, Canada, China, the Netherlands, New Zealand, Norway, the United Kingdom, or the United States. The number of participants in each study ranged between 20 and 1767. Their ages varied in the age range of 11 and 24 years. The attrition rate of the studies was between 0%(0/38) and 67.3% (385/572) (see Multimedia Appendix 2).

#### Meta-Analysis

Fifteen studies (16 hits) included in the meta-analysis were published from 2009 to 2016. The total number of participants was 4979. The attrition rate of the included studies ranged from 0% (0/38) to 61.12% (1080/1767). (see Multimedia Appendix 2).

### Description of the Interventions

In 22 eligible studies (27 articles), a variety of Web-based interventions were used (see Multimedia Appendix 3). The most common background approach used was CBT. Related to materials and procedures, the interventions were composed of modules, sessions, or lessons, with a variety of themes of background theories. ICT-based interventions used interactive games, online chats, mobile phone apps, and emails. The participants were offered activities to support their progress, such as homework assignments or exercises, skill training, workbooks or guided work, quizzes, and questionnaires. Interventions were provided by various professionals such as teachers, school counselors, research team members, project coordinators, or health care personnel. The programs were delivered on websites through computer software via compact disc read-only memory, mobile phone apps, or emails. They were offered at schools, in health care services, or in community settings. The interventions could include up to 14 modules that lasted from 3 to 10 weeks, typically done once per week. The time spent on the programs ranged from 20 min to 3 hours per week. Regarding the fidelity of the intervention, the participants’ completion of the intervention varied between 10% and 94%.

### Risk of Bias in the Meta-Analysis

Most studies (12/15) included in the meta-analysis had a low risk of selection bias in random sequence generation. Half of the studies (7/15) had a low risk in allocation concealment, whereas in 4 studies (4/15), the risk was high. The risk involved with blinding participants and personnel (13/15) and outcome assessment (11/15) was low in most studies. More concern was raised regarding attrition bias (6/15 had a high risk) and especially, selective reporting (8/15 had a high risk). Out of 15 included studies, a published trial registration or a protocol was not found for 3 studies (Figure 2).

### Effectiveness of the Interventions on Depressive Symptoms

For the primary outcome, a meta-analysis was performed involving 10 studies [44,47-51,53-55,57]. We compared Web-based interventions with the control groups of the studies by investigating the short-term effects of the interventions on depressive symptoms. This analysis (postintervention measurement) showed statistically significant improvements in the Web-based intervention groups (*P*=.02, median 1.68, 95% CI 3.11-0.25). However, only 4 of the studies [36,38,40,54] compared the effects on depressive symptoms regarding mid-term effects (follow-up measurements after 3-5 months). No statistically significant improvements in the Web-based intervention group were found in these comparisons (*P*=.08, median 2.91, 95% CI 6.19-0.36).

We further assessed the long-term effects of the Web-based interventions. Out of 10 studies, we found 3 studies [44,49,54] that assessed the long-term effects (6 months or longer). As for short-term effects after intervention, statistically significant improvements were found in adolescents’ depression scores in the Web-based intervention group (*P*=.01, median 1.78, 95% CI 3.20-0.37).

**Figure 3:**
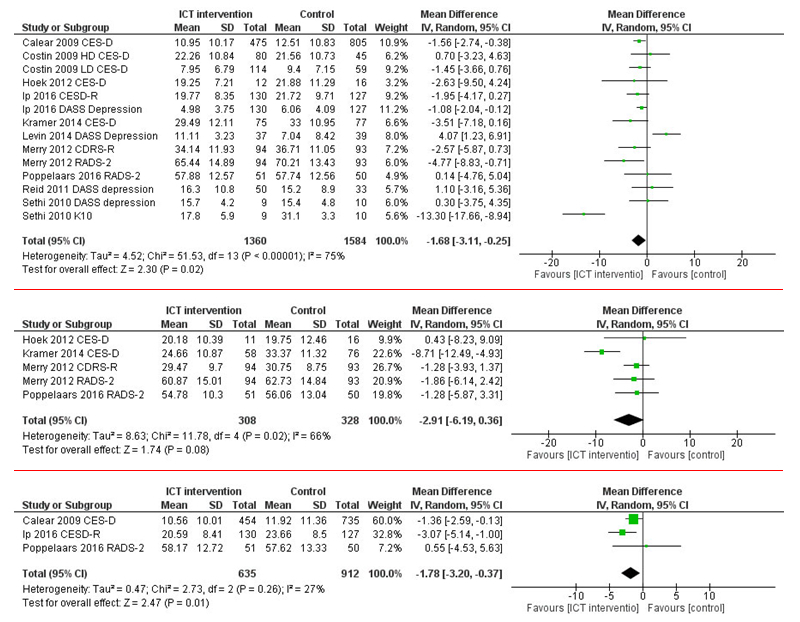
Short-, mid-, and long-term effectiveness of Web-based interventions on depressive symptoms compared with that of a control group.

Substantial heterogeneity was found in the short-term and mid-term effects, but regarding the long-term effects, heterogeneity was only at a moderate level (see Figure 3).

### Web-Based Intervention Group Versus Control Regarding Anxiety Symptoms

**Figure 4:**
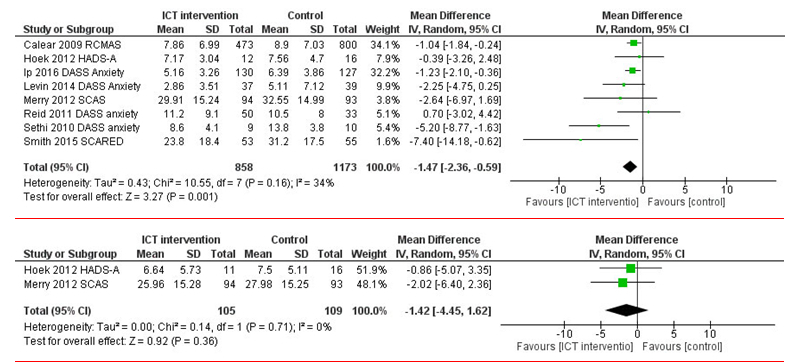
Short-term and mid-term effectiveness of Web-based interventions on anxiety symptoms compared with that of a control group.

Anxiety symptoms were assessed in 8 studies comparing short-term effects of Web-based interventions to control groups [44,48,49,51,53,55,57,58]. Statistically, significant improvements were found in the Web-based intervention group in the short term (*P*=.001, median 1.47, 95% CI 2.36-0.59). However, for the mid-term assessment (follow-up measurements after 3-5 months), only 2 studies evaluated the effectiveness of a Web-based intervention for anxiety symptoms [48,53], and no statistically significant improvements in the symptoms were found (*P*=.36, median 1.42, 95% CI 4.45-1.62; see Figure 4). None of the studies measured the effectiveness at the 6-month mark.

Tests evaluating heterogeneity showed that for short-term and mid-term effects, heterogeneity was on a moderate level (see Figure 4).

**Figure 5:**
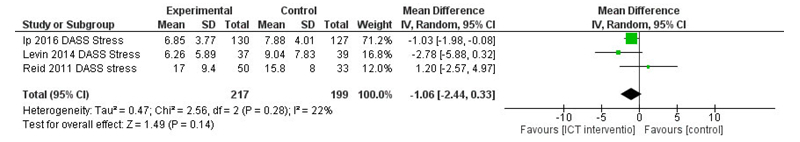
Short-term effectiveness of Web-based interventions on stress symptoms compared with that of a control group.

### Web-Based Intervention Group Versus Control Regarding Stress Symptoms

An analysis of stress outcomes was performed (3 studies) [49,51,55] to compare the effectiveness of a Web-based intervention on stress symptoms with that of a control group. The postintervention comparison showed no statistically significant short-term improvements in the intervention group (*P*=.14, median 1.06, 95% CI 2.44-0.33; see Figure 5).

Heterogeneity tests showed that heterogeneity was on a moderate level in short-term effects (see Figure 5).

### Web-Based Intervention Group Versus Control Regarding Moods and Feelings

**Figure 6:**
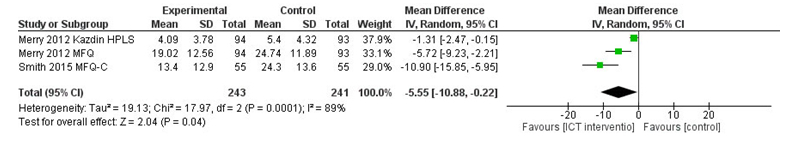
Short-term effectiveness of Web-based interventions on moods and feelings compared with that of a control group.

A meta-analysis was performed on 2 studies [53,58] to compare Web-based interventions with control groups with regard to short-term effects on moods and feelings. These comparisons (postintervention measurement) showed some statistically significant improvements in the Web-based intervention groups (2 studies; *P*=.04, median 5.55, 95% CI 10.88-0.22). Heterogeneity tests showed that heterogeneity was at a considerable level in the short-term effects (see Figure 6).

### Web-Based Intervention Group Versus Control in Leaving the Study Early (Attrition)

**Figure 7:**
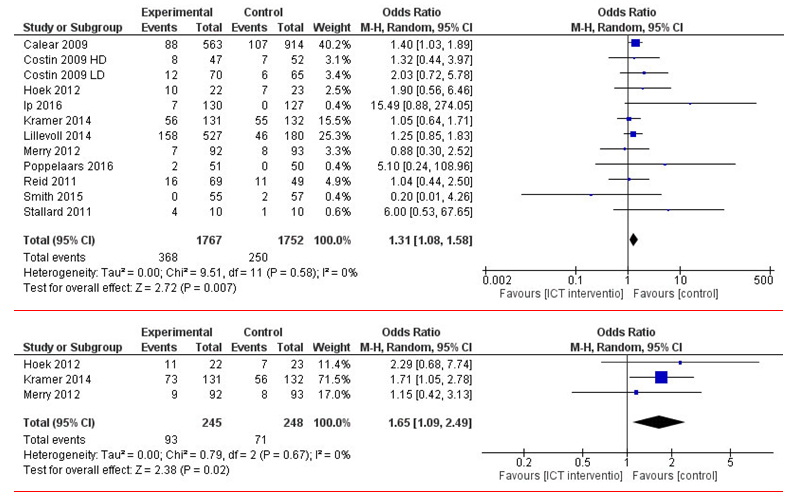
Short- and mid-term effectiveness of Web-based interventions on leaving the study early compared with that of a control group.

Regarding the secondary outcome, leaving the study early, a meta-analysis was performed on 11 studies [44,47-50,52-55,58,59]. Postintervention measurement comparisons of short-term effects on leaving the study early regarding showed statistically significant results favoring the control group (*P*=.007, median 1.31, 95% CI 1.08-1.58). In an assessment of the mid-term effects (follow-up measurements after 3-5 months), 3 studies were compared [48,50,53]. Again, a statistically significant result favored the control group, showing that young people left the study earlier in the intervention group (*P*=.02, median 1.65, 95% CI 1.09-2.49). Heterogeneity tests showed substantial heterogeneity both in short and mid-term effects (Figure 7). An analysis of long-term effects was not possible because of missing data.

## Discussion

### Principal Findings

Our review showed fluctuations in the results of the effectiveness of the Web-based interventions; there was statistically significant improvement in the short-term and long-term (over 6 months with 2 studies) effects regarding adolescent depression but not in mid-term effects. Furthermore, in scores regarding anxiety and moods and feelings, a statistically significant improvement was found after the intervention but not in follow-up measurements. The fluctuation in the depression scores and a lack of significant findings may be a result of the small number of studies that included follow-up measurements. More studies with longer follow-up periods should therefore be conducted to produce clinically significant evidence on the long-term effectiveness of Web-based interventions.

### Comparison With Prior Work

This review is pertinent because the effective interventions for supporting adolescent health are an investment in public health and the future (63). As 92% of adolescents use the Internet daily (5), Web-based interventions could offer solutions to problems in seeking help with depression (1). In line with previous studies related to psychological therapies [61], we assumed that Web-based interventions might have positive effects on stress levels among young people. However, we were unable to fully confirm our hypothesis. Previous studies have found relief in adolescents’ stress, if the intervention included psychological therapies with face-to-face contact [62]. Contrary to personal contact, Web-based interventions are often self-directed [9,44,52] or self-guided [33]. We can, therefore, ask whether the lack of regular human contact produced less-effective results. Indeed, interventions in our study that favored the intervention group included face-to-face guidance, monitoring of engagement, or follow-up telephone calls by teachers and health professionals (eg, [44,46,49]). We also found that participants in the intervention groups left the study early more often, indicating that they may not have been fully engaged in these Web-based interventions [9].

A comparison of costs of stand-alone online interventions and that of personal communication has not been analyzed thoroughly enough. In general, the use of effective interventions for supporting adolescent health has been seen as an investment in the future of public health [63]. The promises of cost savings with the use of information technology could rely on the fact that most adolescents are already frequent Internet users [5], which could save high investment costs. Its features could be translated into health services and offer easy access [1,3], safeguarded anonymity [4], and opportunities to receive help without the fear of stigmatization [6]. However, the Web-based interventions found in our narrative analysis varied greatly, with diverse background approaches, materials and procedures, providers, delivery types, dosages, and lengths of the intervention. Descriptions of interventions have been of poor quality, which has limited the possibility of comparisons, intervention replication, and the usability of study results in practice [24].

Many studies rely on the opportunities of health technologies for better health outcomes and decreased health costs [7,8]. Our review, however, did not reveal any studies assessing the costs of Web-based interventions. This result is noteworthy because huge investments are currently being made in the development of technological solutions for health services. The World Bank [64] has already screened more than 500 mobile health studies and concluded that evidence regarding the best strategies for effectiveness of the interventions and engaging the users in these interventions is still missing. After our review, the knowledge about the impact of the Web-based interventions remains controversial. Therefore, there is a need for a comprehensive impact evaluation that would show the costs and benefits of Web-based technology in the health sector.

In addition to follow-up periods, larger sample sizes and more rigorous study designs could increase the quality of the research. Specific Web-based interventions instead of *packages of intervention* could also provide a more feasible and accurate conception of the factors impacting the outcomes. Furthermore, more studies are needed in the future to gain a deeper understanding of why adolescents are eager to leave the study and why their engagement in information technology interventions is low.

### Limitations

The results of this study should be considered in the light of its limitations. We only included papers from scientific journals that had been written in English, which may have caused relevant studies written in other languages or existing in gray literature to have been left out [65]. Our review is, therefore, biased toward positive results and western countries. Publication bias may have also affected our results. This review may potentially favor results that have been deemed statistically significant. In addition, a number of studies included in the meta-analysis focused on specific outcomes, which may affect the reliability of some results. Moreover, the heterogeneity of our meta-analysis was high (I^2^ ranged between 0% and 89%). Although I^2^ is not a measure for absolute heterogeneity, it may refer to the high variation in some outcomes between studies [66]. Another point to consider is that the interventions used were more like “packages of interventions,” which included many different elements. As an outcome, it may be difficult to identify which specific elements influenced the effectiveness of the interventions. Furthermore, based on the sensitivity analysis, problems pertaining to the heterogeneity of interventions were identified. We must consider that participants or severity of depressive symptoms could have varied greatly among the studies included in the meta-analysis, which jeopardizes the results of the review [67]. All these issues should be taken into consideration when interpreting the results.

### Conclusions

In conclusion, the principal finding of this review supports the evidence that Web-based interventions are effective in the short term in decreasing depressive and anxiety symptoms and improving moods and feelings among adolescents and young people. The review also indicates that adolescents are not fully engaged in using Web-based interventions. Instead of simply stating that “more studies should be done in this area,” we assert that more critical thinking is needed to understand to whom information technology might be useful, which components or characteristics of interventions make it more effective, and what role human contact in conjunction with information technology may play in engaging and supporting young people with mental health concerns. 

## Appendix

Multimedia Appendix 1 Electronic databases, search terms, and number of hits found.

Multimedia Appendix 2 Characteristics of the studies included in the review.

Multimedia Appendix 3 Descriptions of interventions in included studies.

## Abbreviations

CBT: cognitive behavioral therapy
DASS: Depression Anxiety Stress Scales
ICT: information and communication technology
PRISMA: preferred reporting items for systematic reviews and meta-analysis
RCT: randomized controlled trial
TIDieR: template for intervention description and replication
