# Supplementary material for: Web-Based Interventions Supporting Adolescents and Young People With Depressive Symptoms: Systematic Review and Meta-Analysis
Source: JMIR Mhealth Uhealth. 2017 Dec 8;5(12):e180. doi: 10.2196/mhealth.8624 (PMC5741826; doi:10.2196/mhealth.8624)
Supplement: Multimedia Appendix 1 [file mhealth_v5i12e180_app1.pdf]

Table 1. Electronic databases, search terms and a number of hits found (N)

Search 2015		Search 2017	
Database hits, N	Search terms	Database hits, N	Search terms
Medline (N = 708)<br><br>Psychinfo (N = 478)	(ti(adolescen* OR "young people" OR youngster* OR youth* OR teen* OR juvenile*) OR ab(adolescen* OR "young people" OR youngster* OR youth OR teen* OR juvenile*) OR su(adolescen* OR "young people" OR youngster* OR youth* OR teen* OR juvenile*)) AND ((SU.EXACT("Client Education") OR SU.EXACT("Counseling") OR SU.EXACT("Psychotherapeutic Counseling")) OR ti(treatment* OR intervention* OR prevent* OR teaching* OR guidance* OR support* OR therap* OR psychotherap* OR self-manage* OR self-help* OR "patient education*" OR "client education*" OR "patient information*") OR ab(treatment* OR intervention* OR prevent* OR teaching* OR guidance* OR support* OR therap* OR psychotherap* OR self-help* OR self-manage* OR "patient education*" OR "client education*" OR "patient information*") OR SU.EXACT("Self Management") OR SU.EXACT("Self Help Techniques") OR (SU.EXACT("Teaching") OR SU.EXACT("Information"))) AND (ti(outcome* OR effectiv* OR impact* OR design* OR develop* OR protocol*) OR ab(outcome* OR effectiv* OR impact* OR design* OR develop* OR protocol*) OR SU.EXACT.EXPLODE("Treatment Outcomes") OR SU.EXACT("Treatment Effectiveness Evaluation")) AND ((SU.EXACT.EXPLODE("Communication Systems") OR SU.EXACT("Internet") OR SU.EXACT("Computer Assisted Therapy") OR SU.EXACT("Online Therapy") OR	Medline<br>PsycInfo<br>Cochrane<br>(N = 587)	(ti(adolescen* OR "young people" OR youngster* OR youth* OR teen* OR juvenile*) OR ab(adolescen* OR "young people" OR youngster* OR youth OR teen* OR juvenile*) OR su(adolescen* OR "young people" OR youngster* OR youth* OR teen* OR juvenile*)) AND ((SU.EXACT("Client Education") OR SU.EXACT("Counseling") OR SU.EXACT("Psychotherapeutic Counseling")) OR ti(treatment* OR intervention* OR prevent* OR teaching* OR guidance* OR support* OR therap* OR psychotherap* OR self-manage* OR self-help* OR "patient education*" OR "client education*" OR "patient information*") OR ab(treatment* OR intervention* OR prevent* OR teaching* OR guidance* OR support* OR therap* OR psychotherap* OR self-help* OR self-manage* OR "patient education*" OR "client education*" OR "patient information*") OR SU.EXACT("Self Management") OR SU.EXACT("Self Help Techniques") OR (SU.EXACT("Teaching") OR SU.EXACT("Information"))) AND (ti(outcome* OR effectiv* OR impact* OR design* OR develop* OR protocol*) OR ab(outcome* OR effectiv* OR impact* OR design* OR develop* OR protocol*) OR SU.EXACT.EXPLODE("Treatment Outcomes") OR SU.EXACT("Treatment Effectiveness Evaluation")) AND ((SU.EXACT.EXPLODE("Communication Systems") OR SU.EXACT("Internet") OR SU.EXACT("Computer Assisted Therapy") OR SU.EXACT("Online Therapy") OR

	SU.EXACT.EXPLODE("Electronic Communication") OR SU.EXACT("Telecommunications Media")) OR ti(internet* OR web* OR online* OR technolog* OR eHealth OR computer* OR ICT OR smartphone* OR "social media") OR ab(internet* OR web* OR online* OR technolog* OR eHealth OR computer* OR ICT OR smartphone* OR "social media")) AND (ti(depress* OR "mood disorder*" OR "affective disorder*") OR ab(depress* OR "mood disorder*" OR "affective disorder*") OR SU.EXACT.EXPLODE("Major Depression") OR SU.EXACT("Depression (Emotion)") OR SU.EXACT("Affective Disorders"))	SU.EXACT.EXPLODE("Electronic Communication") OR SU.EXACT("Telecommunications Media")) OR ti(internet* OR web* OR online* OR technolog* OR eHealth OR computer* OR ICT OR smartphone* OR "social media") OR ab(internet* OR web* OR online* OR technolog* OR eHealth OR computer* OR ICT OR smartphone* OR "social media")) AND (ti(depress* OR "mood disorder*" OR "affective disorder*") OR ab(depress* OR "mood disorder*" OR "affective disorder*") OR SU.EXACT.EXPLODE("Major Depression") OR SU.EXACT("Depression (Emotion)") OR SU.EXACT("Affective Disorders"))
Cochrane (N = 79)	MeSH descriptor: [Adolescent] explode all trees OR ti(adolescen* or "young people" or youngster* or youth* or teen* or juvenile*:ti (Word variations have been searched)) OR ab(adolescen* or "young people" or youngster* or youth* or teen* or juvenile*:ab (Word variations have been searched) AND MeSH descriptor: [Depressive Disorder, Major] explode all trees OR MeSH descriptor: [Mood Disorders] this term only OR MeSH descriptor: [Depression] explode all trees OR ti(depress* OR "mood disorder*" OR "affective disorder*") OR ab(depress* OR "mood disorder*" OR "affective disorder*") AND ti(outcome* OR effectiv* OR impact* OR design* OR develop* OR protocol*) OR ab(outcome* OR effectiv* OR impact* OR design* OR develop* OR protocol*) OR MeSH descriptor: [Treatment Outcome] this term only OR MeSH descriptor: [Outcome Assessment (Health Care)] this term only AND MeSH descriptor: [Internet] this term only OR MeSH descriptor: [Communications Media] this term only OR ti(internet* OR web* OR online* OR technolog* OR eHealth OR computer* OR ICT OR	

	smartphone* OR "social media") OR ab(internet* OR web* OR online* OR technolog* OR eHealth OR computer* OR ICT OR smartphone* OR "social media") AND MeSH descriptor: [Patient Education as Topic] this term only OR MeSH descriptor: [Counseling] this term only OR MeSH descriptor: [Teaching] this term only OR ti(treatment* or intervention* or prevent* or teaching* or guidance* or support* or therap* or psychotherap* or self-manage* or self-help* or "patient education*" or "client education*" or "patient information*":ti and treatment* or intervention* or prevent* or teaching* or guidance* or support* or therap* or psychotherap* or self-manage* or self-help* or "patient education*" or "client education*" or "patient information*") OR ab(Word variations have been searched for)		
Cinahl (N = 218)	(MH "Adolescence") OR TI (adolescen* OR "young people" OR youngster* OR youth* OR teen* OR juvenile*) OR AB (adolescen* OR "young people" OR youngster* OR youth* OR teen* OR juvenile*) AND (MH "Depression+") OR (MH "Affective Symptoms")) OR TI (depress* OR "mood disorder*" OR "affective disorder*") OR AB (depress* OR "mood disorder*" OR "affective disorder*") AND ((MH "Internet+") OR (MH "World Wide Web+") OR (MH "Telecommunications") OR (MH "Communications Media") OR (MH "Therapy, Computer Assisted") OR (MH "Online Services")) OR TI (internet* OR web* OR online* OR technolog* OR eHealth OR computer* OR ICT OR smartphone* OR "social media") OR AB (internet* OR web* OR online* OR technolog* OR eHealth OR computer* OR ICT OR smartphone* OR "social media") AND (MH "Patient Education") OR (MH "Counseling") OR (MH "Self Care") OR (MH "Teaching+") OR (MH "Health Information	Cinahl (N = 17)	(MH "Adolescence") OR TI (adolescen* OR "young people" OR youngster* OR youth* OR teen* OR juvenile*) OR AB (adolescen* OR "young people" OR youngster* OR youth* OR teen* OR juvenile*) AND (MH "Depression+") OR (MH "Affective Symptoms")) OR TI (depress* OR "mood disorder*" OR "affective disorder*") OR AB (depress* OR "mood disorder*" OR "affective disorder*") AND ((MH "Internet+") OR (MH "World Wide Web+") OR (MH "Telecommunications") OR (MH "Communications Media") OR (MH "Therapy, Computer Assisted") OR (MH "Online Services")) OR TI (internet* OR web* OR online* OR technolog* OR eHealth OR computer* OR ICT OR smartphone* OR "social media") OR AB (internet* OR web* OR online* OR technolog* OR eHealth OR computer* OR ICT OR smartphone* OR "social media") AND (MH "Patient Education") OR (MH "Counseling") OR (MH "Self Care") OR (MH "Teaching+") OR (MH "Health Information

**Total N = 1,483**	Systems")) OR TI (treatment* OR intervention* OR prevent* OR teaching* OR guidance* OR support* OR therap* OR psychotherap* OR self-manage* OR self-help* OR "patient education*" OR "client education*" OR "patient information*") OR AB (treatment* OR intervention* OR prevent* OR teaching* OR guidance* OR support* OR therap* OR psychotherap* OR self-manage* OR self-help* OR "patient education*" OR "client education*" OR "patient information*") AND ((MH "Outcomes (Health Care)") OR (MH "Treatment Outcomes") OR (MH "Outcome Assessment") OR (MH "Health Impact Assessment")) OR TI (outcome* OR effectiv* OR impact* OR design* OR develop* OR protocol*) OR AB (outcome* OR effectiv* OR impact* OR design* OR develop* OR protocol*)	**Total N = 604**	Systems")) OR TI (treatment* OR intervention* OR prevent* OR teaching* OR guidance* OR support* OR therap* OR psychotherap* OR self-manage* OR self-help* OR "patient education*" OR "client education*" OR "patient information*") OR AB (treatment* OR intervention* OR prevent* OR teaching* OR guidance* OR support* OR therap* OR psychotherap* OR self-manage* OR self-help* OR "patient education*" OR "client education*" OR "patient information*") AND ((MH "Outcomes (Health Care)") OR (MH "Treatment Outcomes") OR (MH "Outcome Assessment") OR (MH "Health Impact Assessment")) OR TI (outcome* OR effectiv* OR impact* OR design* OR develop* OR protocol*) OR AB (outcome* OR effectiv* OR impact* OR design* OR develop* OR protocol*)
