# Supplementary material for: Web-Based Interventions Supporting Adolescents and Young People With Depressive Symptoms: Systematic Review and Meta-Analysis
Source: JMIR Mhealth Uhealth. 2017 Dec 8;5(12):e180. doi: 10.2196/mhealth.8624 (PMC5741826; doi:10.2196/mhealth.8624)
Supplement: Multimedia Appendix 2 [file mhealth_v5i12e180_app2.pdf]

Table 3. Characteristics of the studies included in the narrative review rivissa tyhja vali = molemmin puolin

Author<br>Year	Country	Purpose	Setting	Target group<br>Age<br>A number of randomized (N)	Allocation to study groups (n)	Follow-up (n)<br>Attrition %
**Burckhardt et al. 2015**[30]	Australia	To examine the feasibility of an online school-based positive psychology program	High schools	High-school students,<br>12–15 years<br>N=572	1) Bite Back (n=313)<br>2) Control group (n=259)	187<br>67%
**# Calear et al. 2016**[45]	Australia	To test the effectiveness of an online self-directed anxiety prevention program, and to compare two methods of implementing an anxiety program	Schools	30 Schools,<br>Students<br>12–18 years<br>N=1,767	1) eGAD school (n=427)<br>2) eGAD health service (n = 562)<br>3) wait-list control (n = 778)	687<br>61%
**# *Calear et al. 2009**[46]<br>**# *Calear et al. 2013**[47]	Australia	To investigate the effectiveness of an online program in preventing and reducing the symptoms of anxiety and depression and adherence to an internet-based depression prevention program	Schools	32 schools, students<br>12–17 years<br>N=1,477	1) MoodGYM (n = 563)<br>2) Wait-list (n = 914)	1,194<br>19%
**# Costin et al. 2009**[48]	Australia	To evaluate a brief depression information intervention employing health e-cards	Community	Young people<br>19–24 years<br>N=348	1) e-health card and depression information, basic (n = 114)<br>2) e-health card and depression information, enhanced (n = 117)<br>3) e-health card with health information, control (n = 117)	298<br>14%
**Geisner et al. 2015**[31]	USA	To examine the efficacy of web-based personalized feedback on reducing depressed mood and alcohol use	A public university	Students<br>18–24 years<br>N = 331	1) Alcohol only (n = 84)<br>2) Depressed mood only (n = 84)<br>3) Integrated (n = 78)<br>4) Referral only (n = 85)	311<br>(8%)
**# Hoek et al. 2012**[49]	Netherlands	To evaluate the effects of preventive internet-based self-help problem-solving therapy	Variety of settings (via schools, parents' mental health services, internet)	Adolescents<br>12–21 years<br>N=45	1) Internet-based guided self-help intervention (n = 22)<br>2) A wait list (n = 23)	27<br>40%
**# Ip et al. 2016**[50]	China	To evaluate the effectiveness of Grasp the Opportunity in reducing depressive symptoms	Secondary schools	Adolescents<br>13–17 years<br>N=257	1) Grasp the Opportunity (n = 130)<br>2) attention control (n = 127)	250<br>3%

**# Kramer et al. 2014**[51]	Netherlands	To evaluate the effectiveness of an individual chat treatment	Community (via articles, newspapers, banners, websites, Facebook)	Young people<br>12–22 years<br>N=263	1) PratenOnline (n = 131)<br>2) A wait list (n = 132)	136<br>48%
**# Levin et al. 2014**[52]	USA	To examined the feasibility of a prototype web-based acceptance and commitment therapy program for preventing mental health problems	Colleges	Undergraduate first-year college students<br>18–20 years<br>N=76	1) ACT (n = 37)<br>2) A wait list (n = 39)	73<br>4%
**# Lillevoll et al. 2014**[53]	Norway	To evaluate the feasibility and efficacy of disseminating a self-directed internet-based mental health intervention	Senior high schools	Students<br>15–20 years<br>N=707	1) MoodGYM without reminders (n = 176)<br>2) MoodGYM with standard reminders (n = 176)<br>3) MoodGYM with tailored reminders (n = 175)<br>4) Control condition (n = 180)	503<br>29%
**Manicavasagar et al. 2014**[33]	Australia	To explore the feasibility of the online delivery of a youth positive psychology program	Schools and youth organizations	Adolescents<br>12–18 years<br>N=235	1) Bite Back (n = 120)<br>2) Control websites (n = 115)	167<br>29%
**# Merry et al. 2012**[54]	New Zealand	To evaluate whether the computerized CBT intervention reduces depressive symptoms as much or more than treatment as usual	Primary healthcare	Adolescents<br>12–19 years<br>N=187	1) SPARX (n = 94)<br>2) Control group (n = 93)	168<br>10%
**# Poppelaars et al. 2016**[55]	Netherlands	To teste the effectiveness of OVK and SPARX separately, as well as in combination	Secondary education	Girls<br>11–16 years<br>N=208	1) OVK only (n =50)<br>2) SPARX (n = 51)<br>3) OVK and SPARX combined (n = 56)<br>4) Monitoring control condition (n = 51)	159<br>24%
**# Reid et al. 2011**[56]	Australia	To examine the mental health benefits of the mobile type program	Primary care	Patients in general practices<br>12-24 years old<br>N=118	1) The mobile type monitoring intervention (n = 69)<br>2) The comparison program group (n = 49)	86<br>27%
**# Rickhi et al. 2015**[57]	Canada	To evaluate the effectiveness of an online spirituality informed e-mental health intervention on depression severity	Community	Adolescents<br>13–18 years;<br>Young adults<br>19–24 years<br>N=63	1) Study arm<br>a. Younger (n = 18)<br>b. Older (n = 16)<br>2) Wait list<br>a. Younger (n = 13)<br>b. Older (n = 16)	59<br>6%
**# Sethi et al.**	Australia	To assess the efficacy of online	University, the	Students	1) MoodGYM (n = 9)	38

**2010[58]**		therapy in the treatment and prevention of anxiety and depression	Faculty of Health	18–23 years old<br>N=38	2) Face-to-face therapy and MoodGYM (n = 9)<br>3) Face-to-face therapy (n = 10)<br>4) Control without treatment (n = 10)	0%
**# Smith et al. 2015[59]**	United Kingdom	To test the efficacy of Stressbusters, a Computerized-CBT (C-CBT) program, for depression	Secondary schools	Adolescents<br>12–16 years<br>N=11	1) Stressbusters (n=55)<br>2) Wait list (n=57)	8<br>7%
**# Stallard et al. 2011[60]**	United Kingdom	To describe the development of a software package for depression and anxiety, and report preliminary results on feedback and outcomes	Child and Adolescent Mental Health Services	Young people<br>11–16 years old<br>N=20	1) Think, Feel, Do immediately (n = 10)<br>2) Wait list control, intervention after delay (n = 10)	15<br>25%
**Stasiak et al. 2014[35]**	New Zealand	To examine the feasibility, acceptability, and effects of a computerized program for depressed adolescents	Urban high schools	Students<br>13–18 years old<br>N=34	1) Computerized cCBT (n = 17)<br>2) Computer brief psychoeducation program (n = 17)	19<br>44%
***Van Voorhees et al. 2008[36]<br>Hoek et al. 2011[32]<br>Saulsberry et al. 2013[34]<br>Van Voorhees et al. 2009a[37]<br>Van Voorhees et al. 2009b[38]**	USA	To determine which primary care approach is more efficacious in reducing vulnerability in major depressive disorders	Primary care practice sites	Adolescent patients<br>14–21 years old<br>N=83	1) Motivational interview and CATCH-IT (n = 43)<br>2) Brief advice and CATCH-IT (n = 40)	77<br>7%
**Whittaker et al. 2012[39]**	New Zealand	To develop and test novel mobile phone delivery, a depression prevention intervention	High schools	Students<br>13-17 years old<br>N=855	1) Mobile phone program (n = 426)<br>2) Control program with different content of messages (n = 429)	835<br>2%
**Wright et al. 2017[40]**	United Kingdom	To assess the feasibility of delivering an RCT comparing Stressbusters with an attention control	Children and Adolescent Mental Health Services	Adolescents<br>12–18 years old<br>N=91	1) Stressbusters (n=45)<br>2) Websites (n=46)	55<br>46%

* The study results were reported more than one article

The study was included in the meta-analysis
