# Supplementary material for: Web-Based Interventions Supporting Adolescents and Young People With Depressive Symptoms: Systematic Review and Meta-Analysis
Source: JMIR Mhealth Uhealth. 2017 Dec 8;5(12):e180. doi: 10.2196/mhealth.8624 (PMC5741826; doi:10.2196/mhealth.8624)
Supplement: Multimedia Appendix 3 [file mhealth_v5i12e180_app3.pdf]

Table 4. Descriptions of interventions in included studies

**Author, year**	**Intervention name and theory or rationale**	**Materials and procedures**	**Provider and modes of delivery**	**Location, dose and length of the program**	**Fidelity and use (reason, if available)**
Burckhardt et al. 2015[30]	Bite Back<br><br>Positive psychology (Lyubomirsky 2007)	A workbook, website content, reflective questions. www.biteback.org.au/ Interactive exercises and activities: gratitude entries, mindfulness meditations and exercise (taking photos), personal stories. Comments and answers to questions recorded to workbooks. After session, students emailed completed sections of their workbooks and submitted them to the researchers.	Teachers were responsible for handing workbooks and managing students' behavior during class time. Website pre-moderated by website staff, a senior clinical psychologist advised teachers in challenging situations. Delivered through a structured website.	High schools.<br>A total of 6 hours, 6 weeks (2 schools participated over a 4-week period).	27 (8%) did not return a work-book; 52 (16%) returned 1-2 workbooks; 70 (21%) returned 3-4 workbooks; 187 (56%) returned 5-6 workbooks. Website navigation, a range of materials and information exposed to unknown.
# Calear et al. 2009[46]<br>#Calear et al. 2013[47]	Mood-GYM<br><br>Cognitive behavioral therapy (referred to specific articles)	Module based intervention. Information, animated demonstrations, quizzes, homework. www.moodgym.anu.edu.au Users introduced to six distinct characters that form the basis of examples and discussion (each character has a specific way of dealing with stressful situations). Answers to the exercises and quizzes were recorded in the user's personal workbook accessible at any time.	Project coordinators and classroom teachers provided with a manual containing instructions and scripts on how to deliver the program; no other training. Teachers introduced the program to students, helped, guided, supported use, supervised students in the completion of the program, answered incidental questions during completion. Delivered through a website.	Schools (public, private, coeducational, single-sex, metropolitan, rural).<br>5 modules, 1 per week, each 20–40 min, 5 weeks.	347 (62%) completed 3 or more modules;<br>183 (33%) completed all 5 modules.<br>Mean number of modules completed 3.16.<br>15% completed at least 20/29 exercises.<br>Some students were unable to complete due to absence or other school activities.
# Calear et al. 2016[45]	The e-coach Anxiety and Worry program<br><br>Cognitive behavioral therapy	The program consists of psycho-education and evidence-based toolkits for anxiety. Two psychoeducation sessions included information on anxiety signs and symptoms, risk factors, consequences, and medical, psychological and lifestyle treatment. Three toolkits consisted of CBT, relaxation and physical activity.	Classroom teachers and headspace education officers got research manuals including instructions on how to support students in the completion of the program. No other training was required. Teachers supervised students completing the program. The headspace education officers	Schools, years 9–12, no further details.<br>6 sessions, 1 per week, each 30–40 min, 6 weeks.	333 (78%) completed first 2 weeks; 184 (43%) completed at least 4 weeks; 26 (6%) completed 6 weeks regarding e-GAD school method. 489 (87%) completed first 2 weeks; 365 (65%) completed at least 4 weeks; 281 (50%) completed all 6 weeks regarding e-GAD health service method.

		Exercises and homework included.	provided answers to questions and help to those with mental health concerns. Delivered through a self-directed website.		The program automatically re-corded the commencement and completion of each section.
#Costin et al 2009[48]	Health e-cards<br><br>Help-seeking process (referred to a specific article)	An analogue to postcards (personalized emails with the first name, a greeting and directions to click an URL link to view health info). Information (symptoms of depression, a vignette describing experience of depression, where to find evidence-based info and treatment online, used most with 18 to 24-year-olds, encouragement to consult with a health professional, information about GPs, counselors, clinical psychologists, and psychiatrists).	The project coordinator set an automated emails to each participant. The program was delivered through a website, the health e-cards designed to be read as a series.	Community.<br>3 personalized emails, 1 per week, 3 weeks.	28 (8%) visited no health e-card site;<br>320 (92%) visited at least one site;<br>51 (15%) visited one site;<br>102 (29%) visited two sites;<br>167 (48%) visited all three sites.<br>Adherence to delivered materials was possible to track by analyzing the web logs for each health e-card site.
Geisner et al. 2015[31]	Brief web-based intervention<br><br>Tailored social norms approach composed of a personalized feedback component (no specific ref. to the theory)	Info and personalized online feedback utilizing text and graphics. Feedback was linked to the screening and baseline surveys; alcohol use and consequences, protective behaviors (i.e. tips/strategies for reducing problematic alcohol use), perceptions, symptoms, coping strategies, and psychoeducation.<br>Additional strategies were listed with encouragement to consider new strategies. Finished when providing a referral list of treatment resources.	E-mail invitations to participate, interested students logged in to the study. Delivered through a website.	Public university.<br>Weekly emails,<br>5 weeks.	137 (89%) reported reading the mood-related intervention materials<br>139 (91%) red the alcohol-related intervention materials.
#Hoek et al. 2012[49]	Internet-based Problem-Solving Therapy<br><br>Problem-Solving Therapy (referred to specific articles)	Consisted of 3 steps: respondents 1) described what really mattered to them, 2) wrote down their current worries and problems, divided problems into categories, and proposed strategies how to solve the problems or to learn to cope with them, 3) made a plan for the future how to accomplish things. Feedback was offered on the completed exercises; directed at mastering the	Professionals provided support. Automated emails were sent to participants to explain the contents and exercises for the coming week. Support to guide the participant through the intervention (an email if the coach does not receive exercises). Delivered through a website.	General population.<br>1 lesson per week,<br>5 weeks.	21 (95%) completed the first lesson;<br>10 (45%) completed three or more lessons;<br>6 (27%) completed all 5 lessons;<br>Intervention not implemented precisely in the way intended due to website problems e.g. technical difficulties in the email support module.<br>6 (27%) encountered delayed

		proposed problem-solving. Links to websites for specific types of problems were offered.			feedback.
#Ip et al. 2016[50]	Grasping the Opportunity<br><br>Behavioral activation, cognitive behavioral therapy; program modified from CATCH-IT (referred to specific articles)	Module-based intervention. Website was available where there was internet access.<br>Improvement of negative cognition, reducing negative behaviors, strengthening resiliency, and reinforcing positive behaviors. Examples in the form of stories to illustrate relevant concepts in the modules. Users were asked about the use of cigarettes, alcohol, and soft drugs.	Research team offered technical support. Instructions were offered about registration, usernames and passwords, and how to access the website. Monthly reminders were sent via phone or social media. Timely assistance in crisis was provided in addition to the program. Delivered through a website.	Secondary school. 10 modules. Participants were asked to visit website anytime there was a possibility, no further details.	18 (7%) did not complete any modules;<br>48 (19%) completed 1 module;<br>52 (20%) 2 modules;<br>73 (28%) 3 modules;<br>11 (4%) 4 modules;<br>9 (4%) 5 modules;<br>10 (4%) 6 modules;<br>1 (1%) 7 modules;<br>6 (2%) 8 modules;<br>3 (1%) 9 modules;<br>26 (10%) 10 modules.<br>The total time spent accessing the intervention and completing the modules was automatically recorded.
#Kramer et al. 2014[51]	Praten Online<br><br>Solution-Focused Brief Therapy (referred to specific articles)	Anonymous individual real-time chats with a trained health care professional in a secured chat room. Available on weekdays, (late) nights, and weekends. Shifts the focus away from problem formation and resolution, to partici-pants' future goals, strengths, and resiliencies. At the end of each chat, the participant decides if their intervention goal was reached. If not, a new chat is scheduled with the therapist.	Trained professionals offered the chat sessions. Participants were asked by email to schedule first chat via the website. Confirmation found after logging in to the personal mailbox on the website. No reminders sent to an email address outside for the sake of anonymity. Delivered through a website in a secured chat room.	General population.<br>Max 5 chats; more sessions delivered when needed, at three selected dates.	73 (56%) logged in to the appointment system;<br>76 (58%) did not have any chats;<br>55 (42%) had one or more chats. Mean number of chats 1.36, on average 4.27 weeks between the first and last chat session (range 0–27 weeks).
#Levin et al. 2014[52]	Web-based acceptance and commitment therapy<br><br>Acceptance and commitment therapy (Hayes et al. 2011)	CBT applying acceptance, mindfulness, behavior change, experiential avoidance. Web-based multimedia lessons (2) and supplementary tailored emails. Collects usage data, integrates responses to previous exercises, automated emails. Didactic sections were	Investigators introduced participants to the program. No further details. Afterwards participation was completed through secure website. An automated e-mail informed participants about the next lesson. Delivered through a website.	Schools (universities and colleges). 2 lessons, 3 weeks.	44 (58%) logged in 2 times;<br>17 (22%) logged in 3 times;<br>12 (16%) logged in 4 to 5 times.<br>65 (85%) red e-mails,<br>52 (69%) were engaged in the suggested exercises.<br>70 (92%) completed the program.<br>On average 3.89 days to complete

		followed by examples, interactive exercises, animations, audio narration, text, graphics, worksheets, experiential exercises, interactive assessments. Completion in pre-determined sequence; counted as completing the lesson after viewing the last page.			each lesson.<br>65 (85%) completed both lessons within the designated 3-week intervention.<br>Meeting with investigators in-person may have inflated program engagement.
Lillevoll et al. 2014[53]	Mood-GYM<br><br>Cognitive behavior therapy (referred to specific articles)	Module-based intervention with personal workbook (exercises). “Characters” model thinking, demonstrates the interaction between mood and thinking (animated diagrams, interactive exercises), types of dysfunctional thinking, how to challenge negative thoughts, self-assessment for dysfunctional thoughts, strategies for overcoming dysfunctional thoughts, self-esteem assessments and training, activities creating positive experiences and emotions.	Researcher registered participants in the e-mail software and ensured weekly e-mail dispatch. Automated weekly emails were sent regardless of program use; otherwise the program delivery was unguided. Researchers briefed the students on the study design following completion of the post-intervention phase. Delivered through a self-directed website.	Senior high schools, completed outside school hours during the participant’s own time.<br>5 modules, each 30–45 minutes,<br>No detailed information about weeks used.	45 (9%) logged in to program;<br>212 (40%) reported non-use.<br>No knowledge if students actually received or read the e-mails.<br>Students might have accessed via a separate user name and password vs. those sent by the research team.<br>The number of actual program users remained unkown.
Manica-vasagar et al. 2014[33]	The Bite Back<br><br>Positive psychology (Seligman 2011)	Information, interactive exercises, methods to develop skills, videos, community noticeboards, links to relevant resources, possibility to leave comments, online discussions, the option of contributing personal work, opinions and stories to the website. Reminder emails was sent once a week to encourage ongoing use and engagement with the websites.	E-mail instruction to create a log-in. Self-guided, no face-to-face contact. In cases of duplicate email addresses or participant names found during the sign-up, the first application was retained and duplicates discarded. Pre-moderated with each comment and upload, monitored and approved before becoming available for public viewing. Delivered through a website.	Schools and youth organizations (no further details).<br>1 hour per week,<br>6 weeks.	37 (61%) reported using the website less than 40 minutes per week;<br>6 (10%) between 40–50 minutes;<br>14 (23%) for between 50–60 minutes<br>Usage data heavily relied on participants’ self-reporting.<br>No further details.
#Merry, et al. 2012[54]	SPARX<br><br>Cognitive behavioral therapy (no specific ref. to	Interactive fantasy game (psychoeducation, relaxation, activity scheduling, communication, interpersonal skills, dealing with emotions, problem solving, cognitive	First person instruction on website. Contact with a healthcare professional/school guidance counselor at recruitment. The only input from health professionals was	Primary healthcare (youth clinics, general practices, school-based counseling	69 (86%) completed at least four modules;<br>48 (60%) all 7 modules,<br>50 (62%) most or all of the homework challenges.

	the theory, NIHCE 2005)	restructuring, mindfulness, relapse prevention). The purpose was to put the game into context, provides education, gauges mood, sets and monitors real-life challenges, equivalent to homework. The person chooses an avatar, undertakes challenges to restore the balance in a fantasy world, supplemented by a paper notebook, spaces to add comments.	a phone call after a month. Young people not improving prompted to seek help from referred clinicians. Delivered by CD-ROM, runs on PCs.	services).<br>7 modules, 3 dimensional games,<br>4 to 7 weeks.	Safety data collected each time points, a brief check 1 month after the intervention starts. The results based on self-report.
#Poppelaars et al. 2016[55]	SPARX<br><br>Cognitive behavioral therapy (referred to specific articles)	Interactive fantasy game purposed to play at home. The game consists of CBT principles and practices, educational interactions with a guide. Real-life homework tasks.	Eligibility screened during class time at school. No further details. Delivered by CD-ROM given to participants for play the game at home.	Secondary schools. 7 levels, 1 per week, each 20–40 minutes. No detailed information about weeks used.	Participants completed an average of 6.48 (SD = 1.27) out of 7 levels according to self-report. One participant (2%) did not play the game, another participant (2%) reported completing only the first level. All remaining participants completed at least 3 levels, 79% of participants completed all seven levels. Approximately 0.5 h for SPARX.
#Reid et al. 2011[56]	Version 4 of the mobile-type program<br><br>Mobile Tracking Young People's Experiences (referred to specific articles)	Monitoring daily mood, stress, coping, activities, eating, sleeping, exercise, location, companions, diet, alcohol and cannabis use, transmits summary to GPs via a secure website. Participants borrowed a study mobile phone. Participants monitored themselves using mobile-type program assessing 8 areas of functioning. Prompted to complete an entry by an auditory signal/beep from the mobile phone at random intervals. If no report completed, the phone emitted one reminder signal. Entries timecoded and saved; no triggers between 10pm and 8am. Participants at risk of self-harm or suicide activated the high-risk alert; SMS automatically sent to on-call	General practitioner reviewed self-monitoring data. GPs trained in using the mobile-type website, provided a study manual including procedure, clinical support, continuing professional development quality assurance points. Weekly reminder faxes sent to GPs and phone calls every other week to GPs clinic. Participants met a research assistant within 5 days of referral. A study manual describing research procedure and offering tips. Upon completion, participants reviewed self-	General practices. Requested to complete at least 2 mobile-type entries a day until returning for the medical review from 2 to 4 weeks (ideal monitoring period). Able to complete the program at any time. Each report took from 1 to 3 minutes to complete.	Participants completed an average of 3.3 mobile-type entries each day (range 1–8 per day) and completed the program in one to 34 days with a mean of 14.6 days.

		psychologist/ phone counselor (who would call the person and assess the risk and alert the local community assistance team).	monitoring data with GP on mobile-type website (GPs instructed to add mobile-type to medical care). An individualized written summary report following structured prescriptive guidelines by registered psychologist. Delivered through a mobile phone application.		
#Richie et al. 2015[57]	The LEAP Project<br><br>An online spirituality informed intervention.<br><br>Principles selected through a literature review	Module-based intervention. Supports adolescents in gaining new perspectives and practical strategies managing life challenges. Spiritually informed principles (e.g. forgiveness, gratitude, compassion). Includes graphic designs with a multimedia format; video clips, insights from a medical expert that has helped others, music clips, youth autobiographical stories, offline activities, relaxation techniques, online journal, moderated comment boxes to share thoughts and experiences, extras section with humor, movie and book suggestions to reinforce the teachings; links to depression information.	Professional host presents and guides participants to use the program materials. The host is an award-winning writer, performer and producer. She was selected based on her ability to openly relate her own experience with clinical depression to the program content, contribution to script writing, and overall passion for the project. The study coordinator explains the study details via telephone. Delivered through a website.	Community.<br>8 modules, approximately 2 to 3 hours in a week, 8 weeks.	54 (87%) competed eight modules,<br>3 (5%) completed more than 50% of the modules;<br>5 (8%) completed less than 50% of the modules.
#Sethi et al. 2010[58]	MoodGYM<br><br>Cognitive-behavioral therapy (Beck 1967, 1987), compilation of tools and workbooks (Menzies 1997)	Information, skills teaching (cognitive restructuring, pleasant activity scheduling, interpersonal problem solving), animated demonstrations, quizzes, ‘‘homework’’ sheets, exercises. moodgym.anu.edu.au Participants receive CBT face-to-face (identified and talked what makes the participant depressed/anxious, recognizes negative	Supervising psychologist carries out the program. The intern psychologist regularly meets the supervising therapist, examined participants clinical progress and treatment protocols. Researcher guided participants through the program and was on hand if questions arose. Delivered through a	University (Faculty of Health Sciences).<br>5 sessions,<br>3 weeks.	N/A

		feelings and coping mechanisms associated), participants work at own pace through the online program.	website.		
#Smith et al. 2015[59]	Stressbusters<br><br>Cognitive behavioral therapy (Verduyn et al. 2009, Wood et al. 1996)	Program consists of psychoeducation about depression, behavioral activation, working with negative thoughts, skills for problem solving, improving social skills, relapse prevention. Includes interactive multimedia (animation and videos), homework, printable customized hand-outs (eg. mood monitoring sheets, or facts about bullying, drug use).	Clinical psychologist interviewed possible participants and offered information about the project after a screening in classrooms. A short clip of the Stressbusters program was shown to them. Delivered by CD-ROM on laptop and headphone in an assigned room in school (max five students in the same room).	Secondary schools.<br>8 sessions, each 30–45 min,<br>8 weeks.	47 (86%) completed all 8 sessions; 51 (93%) completed at least half of the sessions offered.
#Stallard et al. 2011[60]	Think, Feel, Do<br><br>Computerized cognitive behavior therapy (referred to specific articles, NICE 2005)	Interactive software package (CD-ROM) based on workbook and multimedia with sounds, photos, graphics, video clips, cartoons and music. Uses narrators to guide the user through the sessions, interactive responses to quizzes, exercises entered into the program. Responses saved so that previous work can be reviewed. At the end of each session, participants given a brief assignment to complete.	Clinician/multidisciplinary team appraised suitability of participants. An information sheet was given, and a visit to each participant's home was arranged for giving information and showing a short video of the program. Facilitated by a psychology assistant, teacher, nurse. Minimal CBT expertise and training required. The facilitator's role was to discuss and elaborate on the program content, provide support, clarify misunderstandings, help to reflect on the material presented, apply the learnt to own experiences. Delivered by CD-ROM to the participants' homes.	Child and adolescent mental health services. For 1 participant delivered at school.<br>6 sessions, each 30–45 minutes, weekly sessions or more frequent. No detailed information about weeks used.	17 (85%) participants completed all 6 sessions.
Stasiak et al. 2014[35]	The Journey<br><br>Cognitive behavioral therapy (referred to specific articles)	Module-based intervention with fantasy games. User selects and names an avatar, follows narrative of a quest, earns points for completing modules, rewarded with a simple mini-game at the	Participants referred by school counselor. Minimal oversight from the school counselors; instructed to make appointments to use the computer, support offered if	High schools.<br>7 modules, each 25–30 minutes,<br>4 to 10 weeks.	According to self-reports 32 (94%) completed the intervention. Most took 4–6 weeks to complete (flexibility allowed around term break and exam time).

		end of each module. Topics link thoughts and actions to feelings, behavioral activation, pleasant activity scheduling, problem solving, cognitive restructuring, relaxation, relapse prevention). Begins with a mood monitor, followed by a quiz to recap previous module messages, agenda setting, interactive exercises, animations, video clips. Modules end with summary of content and homework assignments.	needed. The program has built-in mood monitoring questions including risk of self-harm questions (if endorsed, resulted in a prompt on the computer suggesting the person see counselor for more help). Delivered by CD-ROM and available on a dedicated stand-alone computer within the counseling department at school.		
Voorhees et al. 2008[36]<br>Hoek et al. 2011[32]<br>Saulsberry et al. 2013[34]<br>Van Voorhees et al. 2009a[37]<br>Van Voorhees et al. 2009b[38]	CATCH-IT<br><br>Behavioral Activation and CBT, Interpersonal Psychotherapy techniques, community resiliency concept, Instructional design theory (referred to specific articles and books)	Module-based intervention. Includes learning goals, review, core concept explanation, adolescent stories, skill-building exercises, summary, feedback, reward. Parent workbook available if participant is under 18, for supporting development and resiliency. Reduces behaviors increasing vulnerability for depressive disorders (e.g. procrastination, avoidance, rumination, pessimistic appraisals, indirect communication style) and increases behaviors protecting against depressive disorder (e.g. countering pessimistic thoughts, and strengthening relationship skills).	Physicians trained for 1–2 hours using lecture and example video tapes. Personal physician or primary care physician conducts the interview. A brief advice interview (1–2 min), or motivational interview (5–15 min) with physician, including 3 motivational phone calls from social worker case manager (received training in interview). Delivered through a website.	Primary care practices. 14 modules, no further information.	Extensively revised based on results and feedback from an initial pilot study.<br>1 (1%) suicide attempt (never visited the site, a prior history of self-harm behavior). The Data Safety and Monitoring Board elected to stop enrollment at 84 of intended 96.<br>Very little contamination of the brief advice condition with motivational interview elements.
Whittaker et al. 2012[39]	MEMO: living in a positive space<br><br>CBT, Behavior change theory, development of mobile phone interventions, social cognitive theory, marketing principles (referred	Text message-based. Messages are a mixture of text messages, video messages of adolescents and celebrities, and animated cartoons. Developed from 15 key messages.	The research team instructed students in schools. Text messages were sent for providing instructions about the study, and permission to video messages Delivered through mobile phone text messages, fully automated program.	High schools.<br>2 mobile phone messages per day, 9 weeks.<br>Monthly messages after intervention.	According to the self-completed forms,<br>311 (74%) viewed at least half of the messages;<br>123 (30%) viewed most or all.<br>324 (39%) shared messages (with anyone, not necessarily someone in the study), they mostly shared only a small number (<10) of messages; not considered sufficient enough to have an effect, even if shared with

	to specific articles)				someone in the other group (contamination).
Wright et al. 2016[40]	Stressbusters<br><br>Cognitive behavioral therapy (Smith et al. 2015)	Interactive presentation featuring animations, graphics. Linearly completed sessions building on knowledge gained in previous sessions and tasks carried out at home between sessions. Homework, e.g. mood diary. Compliance measured between sessions. Includes video inserts (case vignettes). Add-ons as written facts about i.e. bullying, drug use. Participants do mood ratings, activity plans and quizzes, which are stored and used throughout the program.	A researcher provided instructions and practical support at the beginning of every session. Primary Mental Health Workers screened the adolescents before the study. Possible for sessions to be completed at school, CAMHS site, GP surgery or community center. Delivered through computer software.	Community and clinical settings. 8 sessions, each 30–45 min, about one session per week, 8 weeks.	28 (62%) participants in the Stressbuster group completed eight sessions. 32 (70%) who completed at least one session continued with the program and completed all eight sessions. 4 (9%) participants did not complete any sessions.

study is included in meta-analysis
